# Human β-Defensin 4 with Non-Native Disulfide Bridges Exhibit Antimicrobial Activity

**DOI:** 10.1371/journal.pone.0119525

**Published:** 2015-03-18

**Authors:** Himanshu Sharma, Ramakrishnan Nagaraj

**Affiliations:** CSIR-Centre for Cellular and Molecular Biology, Hyderabad, Telangana, India; nanyang technological university, SINGAPORE

## Abstract

Human defensins play multiple roles in innate immunity including direct antimicrobial killing and immunomodulatory activity. They have three disulfide bridges which contribute to the stability of three anti-parallel β-strands. The exact role of disulfide bridges and canonical β-structure in the antimicrobial action is not yet fully understood. In this study, we have explored the antimicrobial activity of human β-defensin 4 (HBD4) analogs that differ in the number and connectivity of disulfide bridges. The cysteine framework was similar to the disulfide bridges present in μ-conotoxins, an unrelated class of peptide toxins. All the analogs possessed enhanced antimicrobial potency as compared to native HBD4. Among the analogs, the single disulfide bridged peptide showed maximum potency. However, there were no marked differences in the secondary structure of the analogs. Subtle variations were observed in the localization and membrane interaction of the analogs with bacteria and *Candida albicans*, suggesting a role for disulfide bridges in modulating their antimicrobial action. All analogs accumulated in the cytosol where they can bind to anionic molecules such as nucleic acids which would affect several cellular processes leading to cell death. Our study strongly suggests that native disulfide bridges or the canonical β-strands in defensins have not evolved for maximal activity but they play important roles in determining their antimicrobial potency.

## Introduction

Human defensins are crucial components of innate immunity and have an important role in killing bacteria, fungi and viruses [1–3]. Their involvements in several other physiological functions are increasingly evident such as fertility, development, wound healing and cancer [4]. Defensins are composed of 30–50 amino acids and rich in cationic residues [1,5,6]. Structurally, they fold into three antiparallel β-strands stabilized by three disulfide bridges [7–10]. On the basis of their disulfide connectivity they are classified as α-, β-and θ-defensins [11–13]. In fact, despite considerable variations in length, amino acid composition and net positive charge, β-strands are observed in all α- and β-defensins. Yet, they differ considerably in their antimicrobial activity [6,14–18]. Several investigations have indicated that all the three disulfide bridges are not essential for the activity of defensins [19–22]. Also, shorter analogs spanning only the cationic segment have been shown to exhibit antimicrobial activity [20,23–26]. Hence, it appears that there is considerable structural plasticity with respect to activity in defensins. In our previous study, we have shown that short analogs spanning the N- and C-terminal segments of HBD4 possess activity only when they have at least a single disulfide bridge and activity was greatly enhanced with three disulfide constraints [23]. The role of disulfide bridges or canonical β-strand conformation in modulating antimicrobial activity of defensins is still ambiguous.

There are several peptide toxins with the number of amino acids comparable to defensins but with the disulfide connectivities entirely different from α and β-defensins. These toxins exert their activity by binding to specific receptors. Conotoxins are peptide toxins isolated from the venom of marine molluscs and specifically target a range of ion-channels [27–29]. Similar to defensins, they also have extensive sequence diversity and variable disulfide bridges [28–30]. μ-conotoxins though have three disulfide bridges, do not have distinctive common secondary structural features [31–34]. The cysteine pattern in μ-conotoxin (-CC-C-C-CC-) is different from the cysteine pattern in defensins (-C-C-C-C-CC-) [28,30,34]. Recent investigations on μ-conotoxins reveal that analogs with non-native cysteine connectivity or with less number of disulfide bridges exhibit activity and selectivity without any significant distortion of the three dimensional structure [35–37].

In this study, we have examined the effect of introducing disulfide bridges different from the one in β-defensins, on structure and antimicrobial activity of the human β-defensin HBD4. The peptides generated and investigated are shown in Table 1. The disulfide pattern in μ-conotoxin was chosen, as despite the presence of three disulfide bridges, the toxins do not show a defined common structural motif observed in defensins [31–34]. Our study suggests that native cysteine connectivity and canonical β-strands are not optimized for the maximal activity in HBD4 and possibly other human defensins. Our study also gives an insight into role of disulfide bridges in antimicrobial mechanism of HBD4.

**Table 1:** Primary structures of HBD4 and the analogs with net charge at neutral pH.

Peptide	Sequence	Net Charge
HBD4^**a**^	ELDRI**C** ^**1**^GYGTAR **C** ^**2**^ RKK **C** ^**3**^ RSQEYRIGR **C** ^**4**^PNTYA**C** ^**5**^ **C** ^**6**^LRK	+7
H4-1d^**b**^	EFELDRI**C** ^**1**^_**Acm**_ **C** ^**2**^_**tBu**_GYGTAR **C** ^**3**^ RKKRSQEYRIGR **C** ^**4**^_**tBu**_PNTYA**C** ^**5**^_**Acm**_ **C** ^**6**^LRK	+6
H4-2d	EFELDRI**C** ^**1**^ **C** ^**2**^_**tBu**_GYGTAR **C** ^**3**^ RKKRSQEYRIGR **C** ^**4**^_**tBu**_PNTYA**C** ^**5**^ **C** ^**6**^LRK	+6
H4-3d	EFELDRI**C** ^**1**^ **C** ^**2**^GYGTAR **C** ^**3**^ RKKRSQEYRIGR **C** ^**4**^PNTYA**C** ^**5**^ **C** ^**6**^LRK	+6
H4-1dΔEF	ELDRI**C** ^**1**^_**Acm**_ **C** ^**2**^_**tBu**_GYGTAR **C** ^**3**^ RKKRSQEYRIGR **C** ^**4**^_**tBu**_PNTYA**C** ^**5**^_**Acm**_ **C** ^**6**^LRK	+7
H4-1d[S]	EFELDRI**SS**GYGTAR **C** ^**3**^ RKKRSQEYRIGR **S**PNTYA**SC** ^**6**^LRK	+6
H4-1d[S]ΔEF	ELDRI**SS**GYGTAR **C** ^**3**^ RKKRSQEYRIGR **S**PNTYA**SC** ^**6**^LRK	+7

^a^ The HBD4 sequence corresponds to the commercially obtained synthetic peptide. The additional EF sequence in analogs corresponds to the start of the mature region of preproHBD4 protein sequence [17].

^b^ Disulfide connectivity in H4-1d, H4-1dΔEF, H4-1d[S] and H4-1d[S]ΔEF is C^3^-C^6^. Disulfide connectivities in H4-2d are C^2^-C^4^ and C^3^-C^6^. Disulfide connectivities in HBD4 and H4-3d are C^1^-C^5^, C^2^-C^4^, and C^3^-C^6^. Side chain protecting groups acetamidomethyl (Acm) and tertiary-butyl (tBu) are shown by subscripts adjacent to cysteines respectively. Cationic residues are underlined.

## Materials and Methods

### Reagents

9-Fluorenylmethoxy carbonyl (Fmoc) amino acids were purchased from Novabiocem AG (Switzerland) and Advanced Chemtech (Louisville, KY). Polyethyleneglycol-polystyrene (PEG-PS) resin was obtained from Milipore (Bedford, MA, US), N-hydroxybenzotriazole hydrate (HOBt) and 2-(1H-benzotriazole-1-yl)-1,1,3,3-tetramethyluronium hexafluorophosphate (HBTU) were from Advanced Chemtech (Louisville, KY, US). Phospholipids, 1-palmitoyl-2-oleoyl-sn-glycero-3-phosphotidylethanolamine (PE), 1-palmitoyl-2-oleoyl-sn-glycero-3-phospho-(1-rac-glycerol)(sodium salt) (PG), and rhodamine labelled PE (Rh-PE) were purchased from Avanti Polar Lipids (Alabaster, AL, US). Thioanisole, ethanedithiol, and m-cresol were either from Fluka AG Chemical Corp, Switzerland or Pierce Chemical Company, US. N, N-diisopropyl ethylamine (DIPEA), sodium chloride (NaCl), sodium dihydrogen phosphate (NaH_2_PO_4_), acetonitrile, α-cyano-4-hydroxycinnamic acid (CHCA), trifluoroacetic acid (TFA), and carboxyfluorescein (CF) were obtained from Sigma-Aldrich, India. Dichloromethane (DCM), dimethyl sulfoxide (DMSO), sodium thiosulfate, N, N’-dicyclohexyl carbodiimide (DCC), 4-dimethylaminopyridine (DMAP), and N, N’-diisopropyl ethylamine (DIPEA) were obtained from S. d. fine-chem Ltd (Mumbai, India). N-(3-triethyl ammoniumpropyl)-4-(6- (4(diethylamino) phenylhexatrienyl) pyridinium dibromide) (FM4-64), and propidium iodide (PI), were obtained from Molecular Probes (Eugene, OR, US). Human β-defensin 4 (HBD4) (Code: 4406-s, Lot No. 590707) was purchased from Peptide Institute Inc. (Osaka). All chemical and solvents used were of high purity grade.

### Synthesis, purification and characterization

Peptides were synthesized by solid phase methods using Fmoc chemistry [38] on PEG-PS resin (0.19 mmol/g). The first amino acid was attached to the resin by forming symmetric anhydride of the amino acid using a mixture of DCM (1–2 ml), DCC (10 equivalents) and activating agent DMAP (5 equivalents) in organic solvent DMF. Subsequent amino acids were attached using HBTU and HOBt as an activating agent during *insitu* activation. Similarly, CF labelling was done at the N-terminus of resin bound peptide with a mixture containing CF and activating agents (HOBt and HBTU) [39]. Cleavage of peptides from the resin was carried out using a mixture of TFA, m-cresol, thioanisole, and ethanedithiol in the ratio of 20:2:2:1 (v/v). Peptides were purified by reversed-phase high performance liquid chromatography (RP-HPLC) on eclipse plus C-18 column (Agilent Technologies, CA, US) with the gradient of 5% to 100% solvent B (0.1% TFA in acetonitrile) in 60 mins. The concentration of peptides were determined using tyrosine absorption coefficient of 1280 M^-1^ cm^-1^ at 280 nm and CF labelled peptides were estimated using absorption coefficient of 65000 M^-1^ cm^-1^ at 492 nm.

### Formation of disulfide bridges

Cysteines in H4-1d, H4-2d and H4-3d were linked through directional synthetic approach which involves the orthogonal protecting groups for the cysteines side chains (S1 Fig.). Three types of orthogonal protecting groups: trityl (Trt), acetamidomethyl (Acm) and tertiary-butyl (tBu) were used for protecting cysteine side-chains. Single disulfide bridged peptide H4-1d was formed by removing Trt from C3 and C6. All protecting groups including Trt were removed during peptide cleavage from the resin. However, protecting groups Acm and tBu attached to the four cysteines were left intact. The first disulfide bridge was formed by incubating the peptide at a concentration of 0.5–1 mg/ml in the oxidizing agent DMSO (20% v/v) overnight [40]. The reaction was stopped by diluting the reaction mixture with two volumes of deionized water containing 0.1% TFA. DMSO was dried under vacuum at room temperature. The peptide was reconstituted in water and purified with RP-HPLC.

The peptide with one disulfide bridge was further processed for the formation of H4-2d that has two disulfide bridges. The second disulfide bridge was formed by deprotecting Acm group from C1 and C5 using iodine as deprotecting and oxidizing agent [25]. The single disulfide peptide purified by RP-HPLC was reconstituted in water:acetic acid (4:1) to a concentration of 100 μg/ml. Iodine (20 equivalents to peptide) was added to the reconstituted peptide and the reaction mixture was kept at 37°C for 6–8 hrs. The reaction was stopped by diluting the reaction mixture with two volumes of deionized water. Iodine was removed from the mixture by washing thoroughly with carbon tetrachloride. The reaction mixture was washed until it became colorless. The peptide was dried in speed vacuum concentrator and dissolved in deionized water and then again was purified with RP-HPLC.

Three disulfide linked peptide H4-3d was formed from H4-2d by removing the tBu group from C2 and C4 as described earlier [41] with few modifications. The peptide was dissolved in TFA at a concentration of 100 μg/ml. 500 equivalents (5 μmol) of DMSO and 100 equivalents (2.5 μmol) of anisole was added to the peptide solution which was kept at 40°C for at least 8–10 hrs with gentle agitation. Peptide was precipitated with ice-cold diethyl ether. Purification of the peptide was carried out using RP-HPLC. The molecular masses of all the analogs were verified by electrospray ionization mass spectrometry (ESI-MS) on a Finnigan LTQ mass spectrometer (Thermo Electron Corporation, San Jose, CA, USA). Samples were dissolved in methanol: water: acetic acid (49: 49: 2 v/v) for electrospray analysis. The theoretical and observed m/z values were: H4-1d (Theoretical: 4898.81; Observed: 4900.04), H4-2d (Theoretical: 4756.61; Observed: 4756.89), H4-3d (Theoretical: 4642.41; Observed: 4641.82), H4-1dΔEF (Theoretical: 4622.52; Observed: 4623.34), H4-1d[S] (Theoretical: 4582.17; Observed: 4582.65), and H4-1d[S]ΔEF (Theoretical: 4305.88; Observed: 4306.08). CF labelled peptides were also purified and characterized in a similar manner.

### Antimicrobial activity

The antibacterial activity of peptides was examined against Gram-negative bacteria *Escherichia coli* (MG1655), *Pseudomonas aeruginosa* (NCTC 6750) and Gram-positive bacteria *Staphylococcus aureus* (NCTC 8530) using colony counting assay. Bacteria were sub-cultured on nutrient broth (Difco, Detroit, MI, US) at 37°C to mid-logarithmic phase from an overnight grown culture. The cells were harvested and washed with 10 mM sodium phosphate buffer (pH 7.4). 100 μl of suspension containing 10^6^ cfu/ml was incubated with varying concentration of peptides for 2 hrs at 37°C. The cells were spread on nutrient agar plates and incubated for 14–18 hrs at 37°C. The numbers of colony forming units (cfu) was counted and minimum concentration at which more than 99% of killing observed was taken as lethal concentration (LC). Fungicidal activity against *Candida albicans* (ATCC 18804) was also determined as described for the bacteria. However, fungal cells were grown in yeast extract-peptone-dextrose (YEPD) (Difco, Detroit, MI, US) at 30°C for 24–30 hrs. For the salt sensitivity assay, the cells were kept in a buffer containing a physiological concentration of NaCl (150 mM) before treatment with lethal concentrations of peptides. The effect of loss of proton motif force (pmf) in bacteria and *C*. *albicans* on the antimicrobial activity of the peptides was determined by adding the metabolic inhibitor like carbonyl cyanide m-chlorophenylhydrazone (CCCP) (50 μM) into the cell suspension 20 mins prior to the peptides treatment. The rate of killing with respect to time was determined by plating the peptide treated cells at fixed time intervals during 2 hrs of incubation. Peptide untreated cells were taken as a control. All the experiments were carried out in triplicates and the data are shown here as mean +/- standard deviation calculated from three independent experiments.

### Preparation of lipid vesicles

Vesicles were prepared by drying desired amount of lipids from stock solutions in chloroform/methanol using nitrogen stream and then were kept for 5–6 hrs in vacuum to remove any traces of organic solvents. For preparation of large unilamellar vesicles (LUVs), desiccated lipid film was hydrated in 10 mM sodium phosphate buffer (pH 7.4) and kept at 4°C overnight. Multilamellar vesicles (MLVs) were formed by vortexing lipid suspensions in sodium phosphate buffer for several minutes. MLVs were passed several times through polycarbonate membranes of 200 nm pore size using mini-extruder (Avanti Polar Lipid Inc.) to get LUVs [42]. Homogeneous size distribution around 200 nm was confirmed by dynamic light scattering analysis using a Photocor Complex-dynamic light scattering (DLS) instrument (Photocor Instruments, MD). A laser of wavelength 632.8 nm was used for collecting the data. The data were processed using DynaLS software (V.2.8.3). GUVs doped with 0.1 mol% Rh-PE were prepared using swelling method [43]. The desiccated lipid film was hydrated with 10 mM sodium phosphate buffer, pH 7.4 at 45°C for 15 mins followed by 10 hrs incubation at 60°C to get vesicles.

### Confocal microscopy

The cellular localization in bacteria and fungi was studied by confocal microscopy using CF-labelled HBD4 analogs. *E*. *coli* and *P*. *aeruginosa* were harvested and resuspended in sodium phosphate buffer (10 mM, pH 7.4) to the concentration of 10^7^ cfu/ml. Bacteria were stained with a lipophilic dye FM4-64 which preferentially stains the inner-membrane lipid phosphotidylethanolamine [44] and thus gives an outline of the bacterial morphology. Dye concentration was kept at 3 μM in the bacterial suspension. To reduce the background due to unbound dye in the solution, cells were washed with sodium phosphate buffer (10 mM, pH 7.4) and resuspended in the same buffer. Cells were then treated with CF labelled peptides H4-1d, H4-2d and H4-3d at concentrations of 1 μM, 5 μM and 5 μM respectively. Cell suspension was transferred into chambered coverglass wells (Lab-Tek II, Thermo Fisher Scientific, MA, US) after peptide treatment and observed on a Leica TCS-SP5 or TCS-SP8 ultraspectral confocal microscope (Leica Microsystems, Germany). Images with Z-sections were taken after 5–10 mins of incubation using 100x oil immersion objective. Excitation wavelengths of 488 nm and 543 nm were used for CF-labelled peptides and FM4-64 respectively. In the case of *C*. *albicans*, propidium iodide (PI) which preferentially stains the nucleic acid content of nonviable cells or cells with compromised membrane was used. Excitation wavelength of 543 nm was used for PI. Images with Z-sections were captured after 15–20 mins of incubation of cells (5 X 10^6^ cfu/ml) with CF labeled peptides H4-1d, H4-2d and H4-3d at concentrations of 4 μM, 5 μM and 10 μM respectively. Bright-field images were also captured simultaneously using transmitted light detector. Time-lapse imaging was used for capturing the kinetics of peptide localization. Cells in buffer were mixed with peptides and images with Z-sections were captured immediately for 15–20 mins in case of bacteria and for 30–45 mins in case of *C*. *albicans*. Images were captured with minimum time intervals. The first image was captured immediately after mixing the peptides with cells. Peptide co-localization with the bacterial membrane or with nucleic acids of *C*. *albicans* was examined using analysis software LAS-AF ver2.0 (Leica Microsystems, Germany). A line was drawn across the bacterial or fungal morphology and fluorescence intensity was measured along the line. Confocal imaging of peptide treated GUVs and MLVs composed of PE-PG (7:3) doped with 0.1 mole% rhodamine-PE (Rh-PE) was performed to examine the binding of peptides with model membrane. CF labeled peptides H4-1d (10 μM), H4-2d and H4-3d (40 μM) were added to the vesicles. The concentration used for each peptide was 4X higher than their respective lethal concentrations against *E*. *coli*. Peptide treated GUVs were kept into chambered coverglass wells and left for 30 mins for vesicles to settle. Confocal images were acquired using 488 nm argon ion laser and 543 nm He-Ne laser for CF and Rh respectively. Vacuole diameter in peptide treated and untreated images of *C*. *albicans* was measured using image analyzing software ImageJ (Ver 1.48, NIH, USA). Expanded images of bacteria and fungi were also obtained using crop tool of ImageJ.

### Circular dichroism (CD)

Far-UV CD spectra were recorded on a Jasco J-815 spectropolarimeter in the region of 185 to 250 nm. H4-1d, H4-2d and H4-3d (12.5 μM) were added into 10 mM sodium phosphate buffer (pH 7.4) or lipid vesicles (LUVs) composed of PE-PG (7:3) (peptide-lipid molar ratio of 1:10) just before recording of spectra. All the spectra were recorded in 0.1 cm path length cell using a step size of 0.2 nm, band width of 1 nm and scan rate of 100 nm/min at room temperature. The spectra were recorded by averaging 8 scans and corrected for solvent/buffer contribution. Smoothing of the spectrum was performed using polynomial fitting. The normalized spectra are reported as mean residual ellipticity per residue (θmre) versus wavelength.

## Results

### Peptides design and characterization

The sequences of HBD4 and its disulfide variants are shown in Table 1. The arrangement of cysteine residues (-CC-C-C-CC-) is similar to the arrangement in μ-conotoxins which show conformational flexibility and do not have a three dimensional structure similar to β-defensins [31–34]. The analogs H4-1d, H4-2d and H4-3d were obtained by selective protection of cysteine side-chains. The sequences of H4-1d, H4-2d and H4-3d have the additional sequence EF at the N-terminus. The activity of HBD4 with the EF sequence as well as the sequence with 11 amino acids at the C-terminus has been reported [45]. While a direct comparison of the activities may not be valid as methods for determination of antibacterial activity as well as the strains were different, it appears that the EF sequence is not a crucial determinant of antimicrobial activity. H4-1dΔEF has EF deletion at the N-terminus. H4-1d[S] and H4-1d[S]ΔEF were single disulfide constrained peptides in which C residues were replaced with S except for C3 and C6. These variants were synthesized to examine the effect of side-chain protecting groups (tBu or Acm) on antibacterial activity.

### Antimicrobial activity

The lethal concentrations (LCs) of peptides including native HBD4 against bacteria and minimum fungicidal concentration (MFC) against *C*. *albicans* are shown in Table 2. The single disulfide bridged analog H4-1d killed bacteria and *C*. *albicans* at ≤ 5 μM while two and three disulfide bridged peptides H4-2d and H4-3d showed lower activity and had LC or MFC in the range of 5–12.5 μM. H4-1d was most potent among all the peptides including HBD4. H4-1d showed four-fold higher activity as compared to native HBD4 against *P*. *aeruginosa* and *S*. *aureus* and two fold higher activity against *E*. *coli*. H4-2d and H4-3d were also more active as compared to HBD4 against *P*. *aeruginosa* and *S*. *aureus*. The data shown in Table 2 clearly indicate that the native disulfide connectivity in HBD4 is not essential for exhibiting antimicrobial activity. The LC of the H4-1d[ΔEF] sequence against *E*. *coli* and *S*. *aureus* was 2.5 μM and 3.5 μM respectively similar to H4-1d, confirming that the EF sequence does not modulate antimicrobial activity. Hence, activity of H4-2d and -3d without EF was not examined. The LC values for H4-1d[S] and H4-1d[S]ΔEF where C residues were replaced with S were 3 μM and 5 μM against *E*. *coli* and *S*. *aureus* respectively indicating that C- tBu and C-Acm do not modulate antibacterial activity. Hence, the antimicrobial activity of H4-1dΔEF, H4-1d[S], and H4-1d[S]ΔEF was not investigated in detail.

**Table 2:** Antimicrobial activity of HBD4 and the analogs.

	HBD4	H4-1d	H4-2d	H4-3d
***E*. *coli*** ^***a***^	5 (0.53)	2.5 (0.84)	10 (2.01)	10 (0.46)
***P*. *aeruginosa***	12 (1.35)	3 (0.11)	6.5 (1.09)	5 (0.15)
***S*. *aureus*** ^***b***^	15 (0.47)	3.5 (1.00)	7.5 (0.28)	6 (1.30)
***C*. *albicans***	7.5 (0.91)	5 (0.10)	10 (2.96)	12.5 (0.34)

Values (in μM) are lethal concentration (LC) against bacteria and minimum fungicidal concentration (MFC) against *C*. *albicans*. Values in parenthesis indicate standard deviation.

^a^ LC values for H4-1dΔEF, H4-1d[S] and H4-1d[S]ΔEF against *E*. *coli* are 2.5 μM, 3 μM and 3 μM respectively.

^b^ LC values for H4-1dΔEF, H4-1d[S] and H4-1d[S]ΔEF against *S*. *aureus* are 3.5 μM, 5 μM and 5 μM respectively.

**Fig 1:**
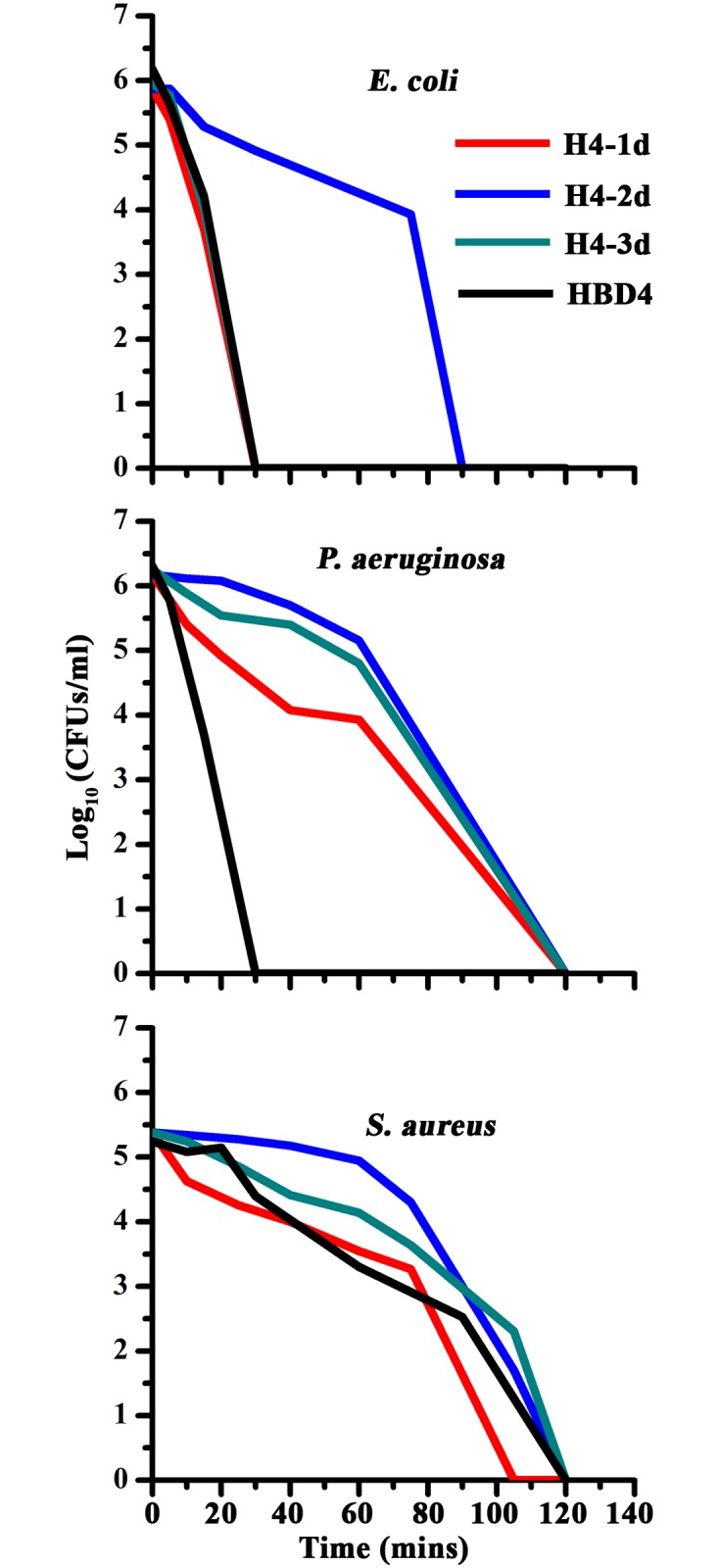
Kinetics of bacterial killing. Kinetics of killing by H4-1d, H4-2d and H4-3d and HBD4.

The kinetics of bacterial killing is shown in Fig. 1. HBD4, H4-1d and H4-3d caused rapid killing of *E*. *coli* within 30 mins of incubation, while killing by H4-2d took almost 80 mins. In the case of *P*. *aeruginosa*, rate of killing by analogs was relatively slower as compared to *E*. *coli*. HBD4 killed *P*. *aeruginosa* more rapidly as compared to the analogs. In the case of *S*. *aureus*, H4-1d showed rapid killing as compared to H4-2d and H4-3d. While the analogs were more active as compared to HBD4, the data indicate that the native disulfide arrangement favors relatively more rapid killing. The requirement of proton motive force (pmf) for activity was also examined. The data presented in S2 Fig. indicate that pmf is not required for antibacterial as well as antifungal activity.

**Fig 2:**
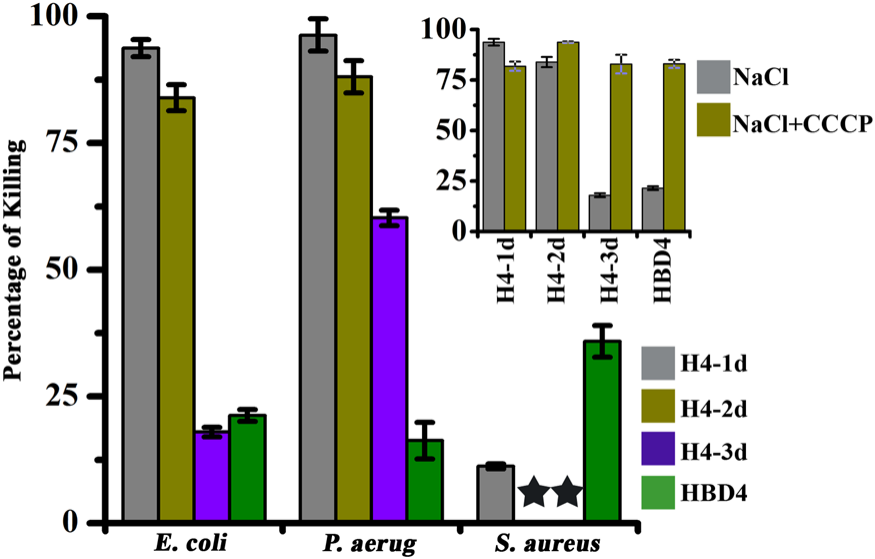
Salt Sensitivity of antimicrobial activity. Effect of physiological salt concentration (150 mM NaCl) on the activity of HBD4 and its analogs. In inset, salt sensitivity of peptides in the presence of the proton motive force (pmf) inhibitor CCCP in case of *E*. *coli* is shown. The symbol star (filled) represents complete loss of activity and *P*. *aerug* represents *P*. *aeruginosa*. The values are average of three independent experiments and the error bars represent standard deviations (range = 0.48–3.58).

The antibacterial activity of HBD1 and 2 is attenuated in the presence of high concentrations of NaCl, whereas the activity of HBD3 remains unaffected [14,16,20]. Since non-native disulfide connectivities in HBD4 enhance antimicrobial activity, we examined the effect of salt on antimicrobial activity of HBD4 and analogs. The data are presented in Fig. 2. H4-1d was active against Gram-negative bacteria *E*. *coli* and *P*. *aeruginosa*. However, activity was lost to a considerable extent against *S*. *aureus*. H4-2d also showed considerable activity against *E*. *coli* and *P*. *aeruginosa* but not against *S*. *aureus*. Loss in activity was observed for H4-3d against *E*. *coli* and *S*. *aureus*. Similarly, HBD4 showed significant loss in activity against *E*. *coli*, *P*. *aeruginosa* and *S*. *aureus*. The results indicate that when three disulfide bridges are present in native as well as non-native configuration, there is considerable attenuation of antibacterial activity at high salt concentration. However, in the absence of proton motive force (pmf) due to protonophore CCCP, attenuation of activity by NaCl is not observed for H4-3d and HBD4 (inset in Fig. 2).

Despite of variation in number and arrangement of disulfide bridges, the peptides possess potent antimicrobial activity. However variation in their potency, rate of killing and salt-sensitivity suggest that the arrangement of disulfide bridges is important for antimicrobial activity.

### Antimicrobial action of peptides examined by fluorescence microscopy

**Fig 3:**
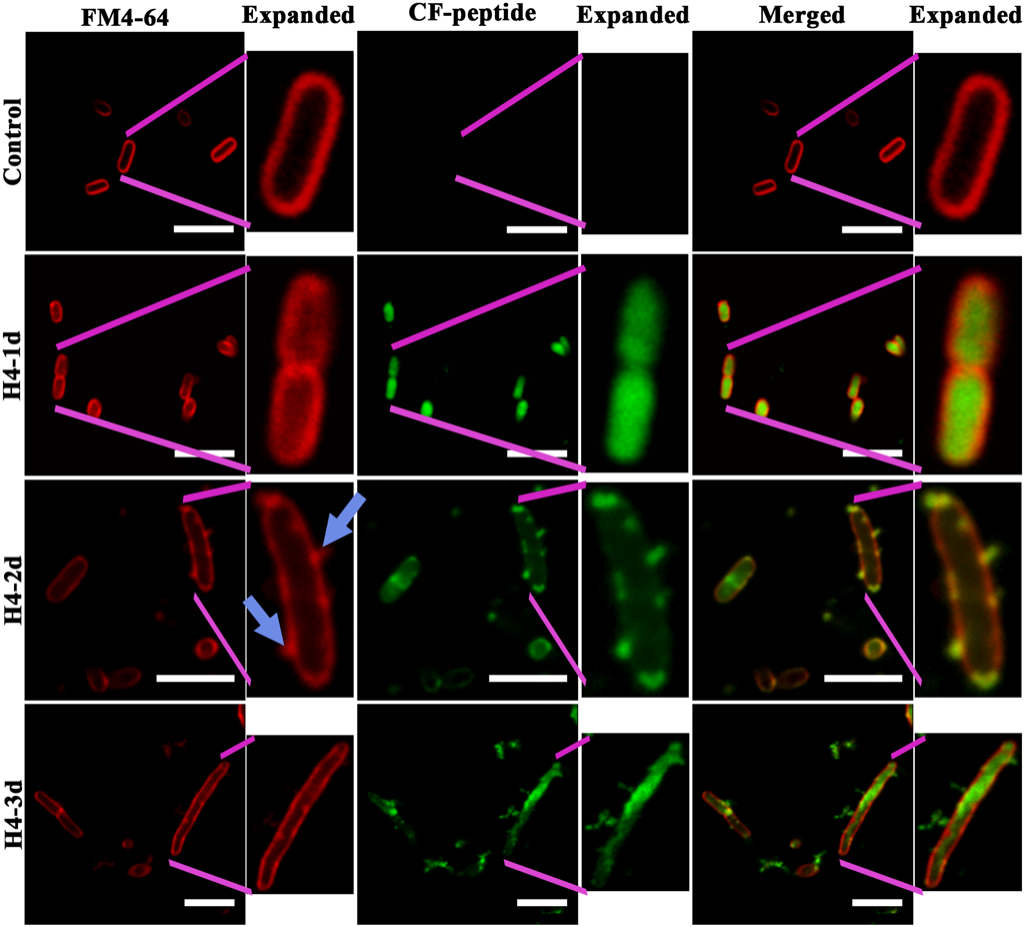
Confocal images of *E*. *coli* on treatment with HBD4 analogs. Localization of caboxyfluorescein (CF) labelled peptides H4-1d, H4-2d and H4-3d in *E*. *coli* stained with inner-membrane dye FM4-64. Expanded images for bacteria are also shown adjacent to each panel indicated by pink lines. Blue arrows in FM4-64 expanded panel show membrane protrusion and lipid aggregation due to H4-2d. Scale bars represent 5 μm.

**Fig 4:**
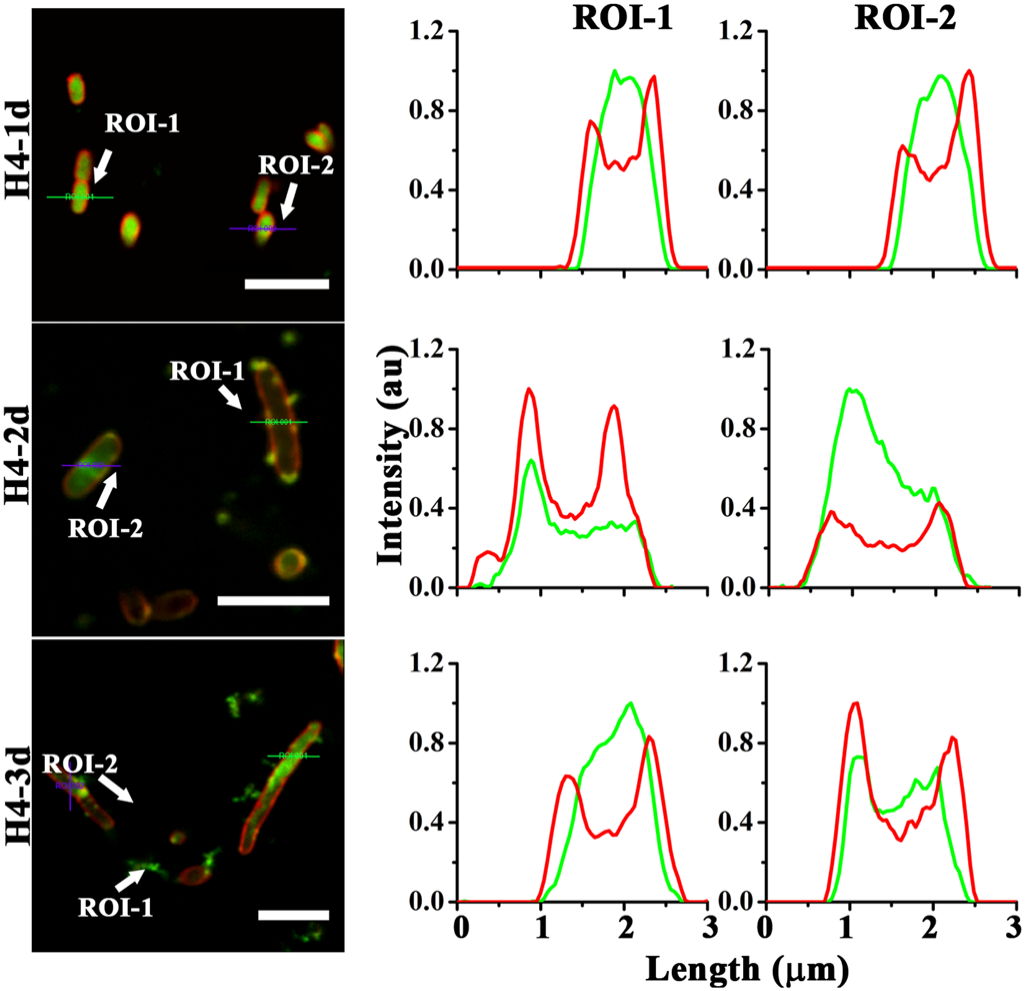
Localization of analogs in *E*. *coli*. Fluorescence intensity due to CF-peptide and FM4-64, represented by green and red color respectively was monitored along the line drawn across the bacterial morphology indicated by white arrows and signifies as region of interest (ROIs). Scale bars represent 5 μm.

To get an insight into the mechanism of action, we have examined the localization of peptides in bacteria and *C*. *albicans* using CF labelled analogs. Antimicrobial activity of the labelled peptides was examined and was found comparable to their respective unlabelled counterparts (data not shown). H4-1d translocated across the *E*. *coli* membrane and accumulated inside the bacteria (Fig. 3). The FM4-64 panel in images of bacteria treated with H4-1d showed diffused staining indicating disruption of inner-membrane integrity. In the case of H4-2d, membrane thinning and aggregation of phospholipid moieties is more prominent as indicated by faint staining and dot like structures on the membrane indicated by blue arrows. H4-3d was localized in the cytoplasm as well as over the membrane, as observed by peptide overlap with membrane staining (Fig. 3). Permeabilization of the inner-membrane on *P*. *aeruginosa* by H4-1-3d is also evident (S3 Fig.). Extent of co-localization of CF-labelled peptides and FM4-64 indicates localization in the cytoplasm for all the peptides (Fig. 4).

**Fig 5:**
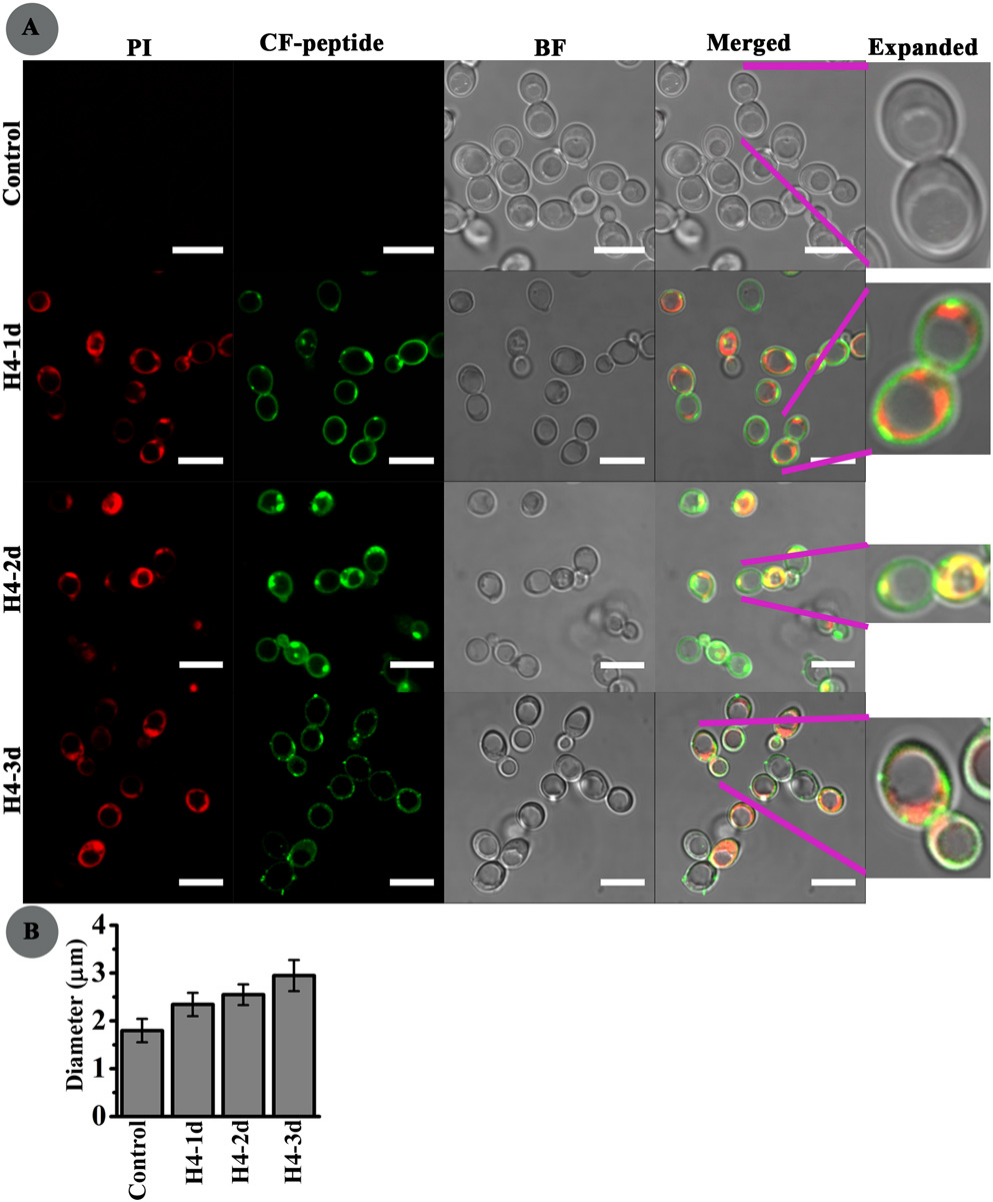
Confocal images of *C*. *albicans* on treatment with HBD4 analogs. **(A)** Localization of carboxyfluorescein (CF) labelled peptides H4-1d, H4-2d, and H4-3d in *C*. *albicans* pre-incubated with nucleic acid stain propidium iodide (PI). The CF-peptides are shown by green fluorescence and nucleic acid is shown by red fluorescence due to PI. Expanded images are shown adjacent to each panel indicated by pink lines. Scale bars represent 7.5 μm. **(B)** Vacuole size. Average size of vacuole diameter (μm) of *C*. *albicans* with or without peptide treatment.

*C*. *albicans* was treated with CF-labelled H4-1d, H4-2d and H4-3d to observe their localization. Before peptide treatment, cells were treated with nucleic acid stain propidium iodide (PI) which internalizes into dead cells or cells with compromised membrane and binds to nucleic acid. Confocal images were captured after 15 mins of incubation. Unlike in bacteria, H4-1d did not spread inside the cytoplasm (Fig. 5A) but accumulated near the cell periphery. PI uptake into the cell indicated disruption of membrane integrity. H4-2d bound to the *C*. *albicans* surface and also spread throughout the cytoplasm. The three disulfide bridged peptide H4-3d formed dot-like aggregates near the membrane periphery which could be the entry point for internalization.

The vacuole size from the images of treated and untreated *C*. *albicans* was determined using image analysis software ImageJ (Version 1.48). Average value for vacuolar diameter, depicted in Fig. 5B, was calculated by measuring vacuoles of several cells from different fields. It was observed that peptides caused 30–60% increase in vacuole size. Cells treated with H4-3d showed maximum increase. Increase in size of vacuoles indicates the loss of cytosolic ions due to peptide treatment. Although, analogs have differences in their cellular localization but it appears that they have common intracellular effects which lead to vacuole expansion.

**Fig 6:**
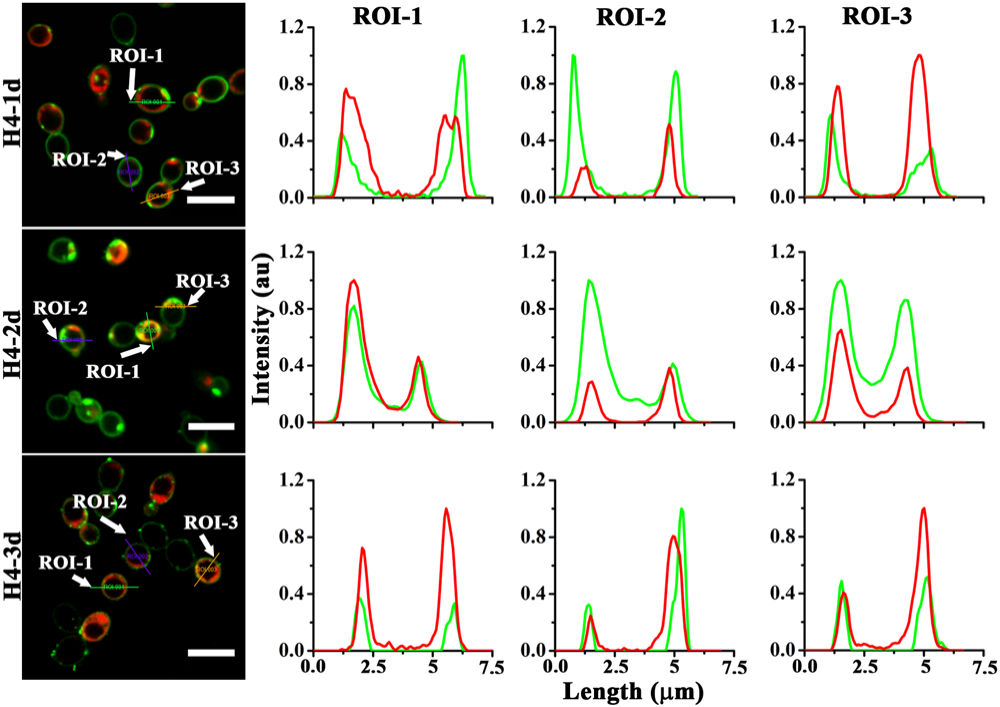
Localization of analogs in *C*. *albicans*. Fluorescence intensity due to CF-peptide and propidium iodide (PI), represented by green and red color respectively, was monitored along the line (ROI) drawn across the bacterial morphology. Scale bars represent 7.5 μm.

Extent of co-localization of CF-labelled peptides and PI are shown in Fig. 6. The data indicated that H4-1d partially co-localized with nucleic acid stain but largely remained near the cell periphery. Fluorescence intensity due to PI and CF-peptide along the line drawn across the *candidal* morphology indicated that H4-2d co-localized with nucleic acid. In some cells, H4-3d remained towards the cellular periphery, whereas in some cells H4-3d partially co-localized with nucleic acid stain.

**Fig 7:**
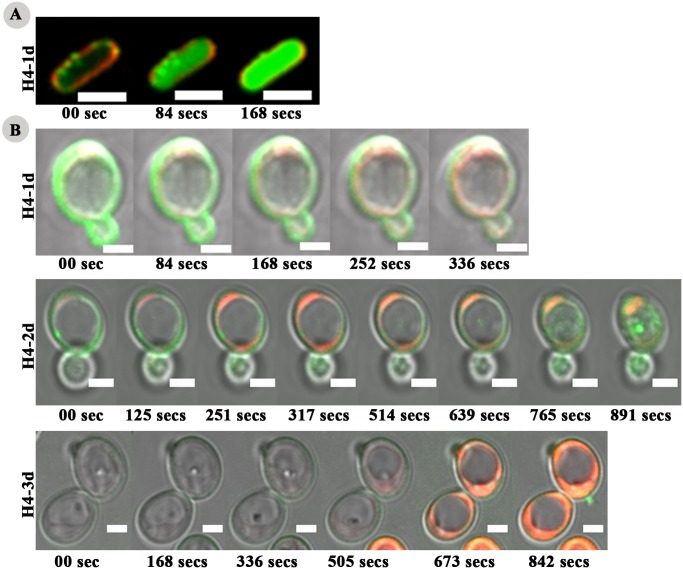
Time lapse images of peptide-microbe interaction. Time-lapse confocal images of *E*. *coli* **(A)** and *C*. *albicans* **(B)** treated with the analogs. Scale bars represent 2 μm.

Further, time-lapse confocal microscopy was performed in order to capture the intermediate stages of internalization. Time-lapse images for the H4-1d in *E*. *coli* are shown in Fig. 7A. H4-1d first binds to the membrane and then translocates across the membrane and accumulates within the cell. Internalization occurred within 2–3 minutes of incubation. Membranes did not show any sign of disruption on peptide binding, but membrane staining became very faint after peptide internalization indicating membrane disruption. In the case of H4-2d and H4-3d, it was not possible to capture the intermediate frames as internalization occurred during the time of peptide mixing with the cell suspension.

In the case of *C*. *albicans* also, images were immediately captured after the peptide treatment with minimum time difference between two time points (Fig. 7B). Time-lapse confocal images showed that H4-1d localized on the surface immediately after peptide added to cell suspension and then with the passage of time, peptide internalized into cell and accumulated near the cell periphery and thereafter PI uptake was occurred within 3–5 mins. However, the two disulfide bridged peptide H4-2d showed membrane binding initially but spread throughout the cytoplasm within 8–10 mins of incubation. Peptide also internalized into vacuole after 12–14 mins of incubation. PI uptake was observed within 5 min of peptide binding. In case of H4-3d also, cytoplasmic localization of peptide occurred within 8–12 mins but initially for 5–8 mins peptide showed very faint staining near the cell periphery. PI uptake occurred within 8–10 mins of incubation. Also, increase in vacuole size is clearly represented by time-lapse images of H4-3d treated cells which was not obvious from images of H4-1d and H4-2d treated cells. The vacuole covered almost the whole surface area within 10–12 mins of incubation. Among the analogs, the peptide with one disulfide bridged showed fastest rate of internalization. Clearly, number of disulfide bridges modulates the rate of internalization and membrane disruption. The data also suggest that vacuole expansion or membrane permeabilization indicated by PI uptake are the consequences of peptide internalization.

### Interaction of peptides with model membranes

**Fig 8:**
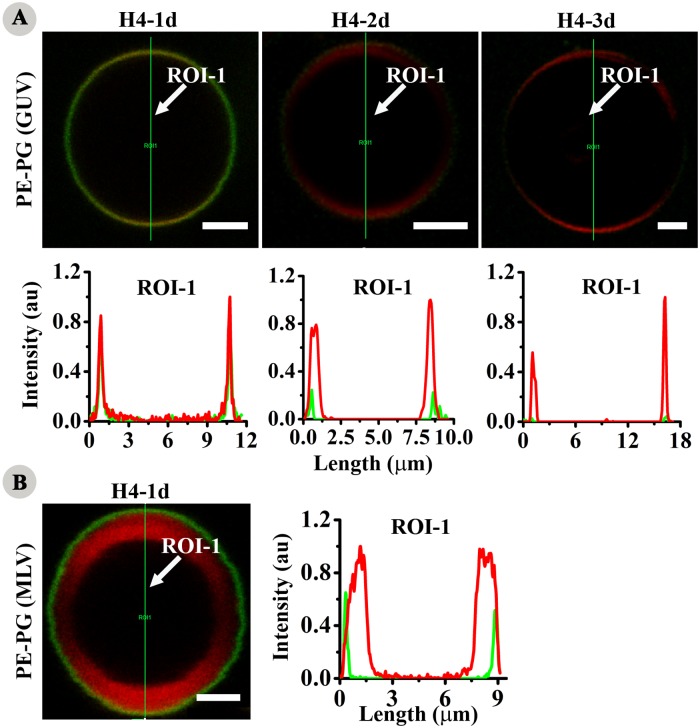
Interaction with lipid vesicles. Localization of CF-labelled analogs with anionic giant unilamellar vesicles (GUVs) and multilamellar vesicles (MLVs) composed of lipids PE and PG in the molar ratio of 7:3 doped with Rh-PE. Green and red fluorescence represent CF-labelled peptide and rhodamine labelled vesicles respectively. Intensity vs length spectra show fluorescence intensity along the lines (ROI-1) drawn across the respective vesicles. **(A)** localization in GUV and **(B)** localization in MLV. Scale bars represent 2.5 μm.

The interaction of defensins with model membranes has been investigated in order to assess the relevance of peptide-lipid interaction with respect to antimicrobial activity [46–48]. Hence, the interaction of CF-labelled analogs of HBD4 with GUV was investigated. Giant unilamellar vesicles (GUVs) and multilamellar vesicles (MLVs) composed of lipid PE and PG in the ratio of 7:3 as a model for representing bacterial membranes doped with Rh-PE were used for localization studies [49,50]. CF-labelled H4-1d did not internalize into the vesicles even at four times higher concentration than LC and after 30 mins of incubation (Fig. 8A). H4-1d co-localized with membrane of the vesicles. This was also evident from the fluorescence intensity along the line drawn across the vesicle morphology. H4-2d and H4-3d also co-localized with the vesicle membrane but showed very faint fluorescence intensity indicating weak binding (Fig. 8A). H4-1d also did not cross multiple bilayers in case of MLVs and was bound to the outermost bilayer of the vesicles (Fig. 8B). No internalization into the vesicles was observed for the analogs. The results suggest that internalization of peptides into the bacteria or *C*. *albicans* is not a simple diffusion process. Peptides did not show any sign of thinning or clustering of lipid moieties of the vesicle membrane indicating membrane defects are specific for the microorganisms.

### Secondary structure

**Fig 9:**
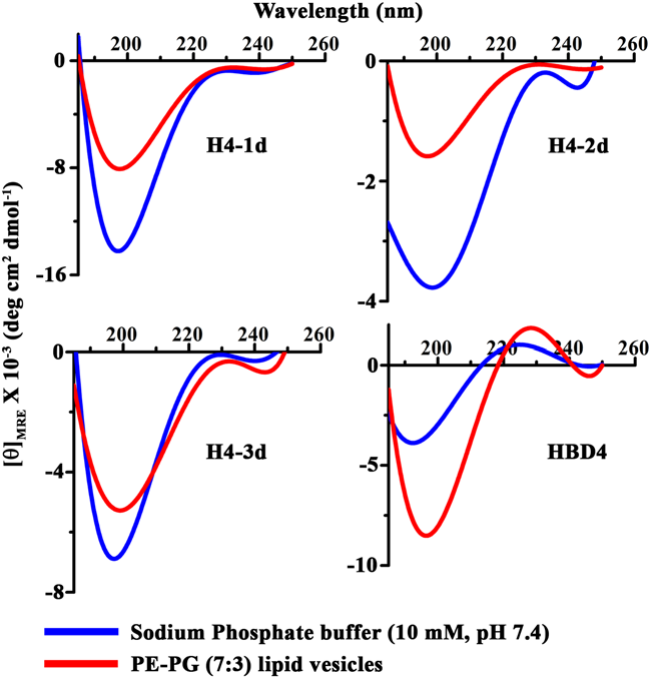
Circular dichroism. CD spectra of HBD4 and its analogs in buffer and lipid vesicles composed of PE-PG (7:3). Peptide-lipid ratio maintained as 1:10.

Circular dichroism (CD) spectra of HBD4 analogs were recorded in buffer and in the presence of lipid vesicles composed of PE-PG (7:3) (Fig. 9). A single minimum is observed for the peptides in buffer and in the presence of lipid vesicles in the region 190–198 nm, indicating that the peptides show flexible conformation. Lipid vesicles do not induce ordered conformation. Qualitatively, variations in the mean residue ellipticities suggest subtle conformational differences between the peptides.

## Discussion

The disulfide connectivities in α- and β-defensins and the common structural motif of three β-strands would suggest that these features are crucial for exhibiting antimicrobial activity [7,10,51]. This argument is largely based on the interactions or proximity of residues observed in the X-ray and NMR structure of defensins [7,10,52,53]. However, peptides spanning the N- and C-terminal segments of HBD1-4 show antimicrobial activity [21,23,25,26]. Also, many of these peptides have only a single disulfide bridge. Activity has also been observed even in some linearized defensins [20,21,25,54]. The reduced form of α- and β-defensins on oxidation gives multiple products with native and non-native disulfide connectivity [19,20,55] indicating that the spacing of cysteines in α- and β-defensins does not appear to have evolved for the formation of a unique disulfide pattern. All of these observations argue against the requirement for the three dimensional structure observed in native defensins for antimicrobial activity. The presence of three disulfide bridges does not necessarily lead to highly ordered structures as observed with certain classes of toxins such as μ-conotoxin [29,30,34]. Recent investigations on μ-conotoxins indicate that non-native cysteine connectivity or breakage of disulfide bridges do not affect the activity and specificity of the peptides. However, in some of the cases, change in binding kinetics or potency to their target was observed [35,37,56]. Therefore, the consequences of engineering the cysteine motif in HBD4 sequence (-C-C-C-C-CC-) by introducing μ-conotoxin motif (-CC-C-C-CC-) on the conformation and the activity was examined to get more and possibly newer structural and mechanistic insights into the folding and activity of defensins. Our investigation suggests that the number of disulfide bridges and the cysteine spacing have a significant effect on the antimicrobial potency.

The peptide with a single disulfide bridge is the most potent among the analogs. When three non-native disulfide bridges are present, greater potency against *P*. *aeruginosa* and *S*. *aureus* but lower activity against *E*. *coli* and *C*. *albicans* is observed as compared to native HBD4. It is thus clearly evident that the arrangement of disulfide bridges as observed in HBD4, does not confer optimal antimicrobial activity. The variations in activity conceivably arise due to differences in the orientation of amino acids, particularly the cationic residues in the different analogs. It has been argued that cationic residues like arginines are capable of strong electrostatic interaction with multiple lipid head groups due to charge delocalization over the guanidinium group [57–59]. Though, highly structured, several defensins show CD spectra [60,61] similar to those shown in our study. The presence of three β-strands in hairpin configuration and N-terminal helix is not evident from the CD spectra. While extensive differences in the secondary structure cannot be inferred from the CD spectra and would require analysis by nuclear magnetic resonance (NMR) spectroscopy, our results suggest that the structural constraints due to variation in the disulfide connectivity could modulate the side-chain conformation of the cationic residues in a manner that strengthen electrostatic interactions with the negatively charged microbial cell surface.

Several linear antimicrobial peptides exert their activity by permeabilizing membranes [62–64]. Even though the sizes of the HBD4 peptides would not permit passage via porins present in the *E*. *coli* outer-membrane, the CF-labelled peptides enter bacterial as well as fungal cells rapidly. We propose that all the three analogs exert their activity by destabilizing the outer-membrane in Gram-negative bacteria via electrostatic interactions and then peptides translocate to the inner-membrane surface which they destabilize but do not lyse. Destabilization of the outer-membrane would be necessary for the peptides to translocate to the inner-membrane as shown for HNP-1 [65]. Also, the membrane defects caused by the different analogs are not similar indicating that the disulfide arrangement plays role in modulating interactions with the membrane surface. X-ray and other studies on α-defensins indicate that they aggregate in membranes to form pores [7,48,66]. The structure of β-defensins HBD1 and HBD2 [67,68] do not suggest such a mechanism. Recently, HBD3 and HNP1 have been shown to inhibit cell wall biosynthesis by binding to lipid II molecules [69,70]. Cationic antimicrobial peptides have been shown to kill pathogens by targeting intracellular components [71–74]. A recent study on magainin-resistant *E*. *coli* strains, which is presumed to act on the bacterial membrane, indicates that the response is complex and several metabolic pathways are involved [75]. The analogs of HBD4 described in this study, perturb the inner-membrane structure which results in the dysfunction of respiratory enzymes. They also enter the cytosol where their presence would affect several metabolic processes. Thus, multiple targets are likely to be involved in the killing of *E*. coli and possibly other microbes.

## Conclusions

In conclusion, our study clearly indicates that native cysteine connectivity and spacing between cysteines in defensins are not evolutionary optimized for maximal activity. Analogs with greater potency as compared to native HBD4 and possibly other β-defensins can be obtained by non-native disulfide connectivities. Permeabilization of bacterial or fungal membranes and cytoplasmic accumulation indicate that killing by HBD4 and possibly by other defensins is not a single event but is a result of multiple events. The mechanism of action is thus different from amphipathic cationic antimicrobial peptides. This study provides important insights into the structure-function relationships in defensins.

## Supporting Information

S1 Fig Disulfide formation. Formation of three disulfide linkages in HBD4 analogs H4-1d, H4-2d and H4-3d with regioselective strategy. Keys: trityl (Trt), acetamidomethyl (Acm), tertiary-butyl (tBu), ethanedithiol (EDT), thioanisole (TA), acetic acid (AcOH.)
(TIF)

S2 Fig Effect of dissipating proton motive force (pmf) by CCCP on antimicrobial activity. Antibacterial and antifungal activity of H4-1d, H4-2d, H4-3d and HBD4 in the presence and absence of CCCP. The values are expressed as average of three independent experiments and the error bars represent standard deviations (range = 0.10–2.96).
(TIF)

S3 Fig Confocal images of *P*. *aeruginosa* on treatment with HBD4 analogs. Localization of carboxyfluorescein (CF) labelled peptides H4-1d, H4-2d and H4-3d in *P*. *aeruginosa* stained with inner-memabrane dye FM4-64. Expanded images for bacteria are also shown adjacent to each panel indicated by pink lines. Blue arrows in FM4-64 expanded panel show membrane protrusion and lipid aggregation due to H4-2d. Scale bars represent 7.5 μm.
(TIF)
