# Supplementary figures and images for: Human β-Defensin 4 with Non-Native Disulfide Bridges Exhibit Antimicrobial Activity
Source: PLoS One. 2015 Mar 18;10(3):e0119525. doi: 10.1371/journal.pone.0119525 (PMC4364940; doi:10.1371/journal.pone.0119525)

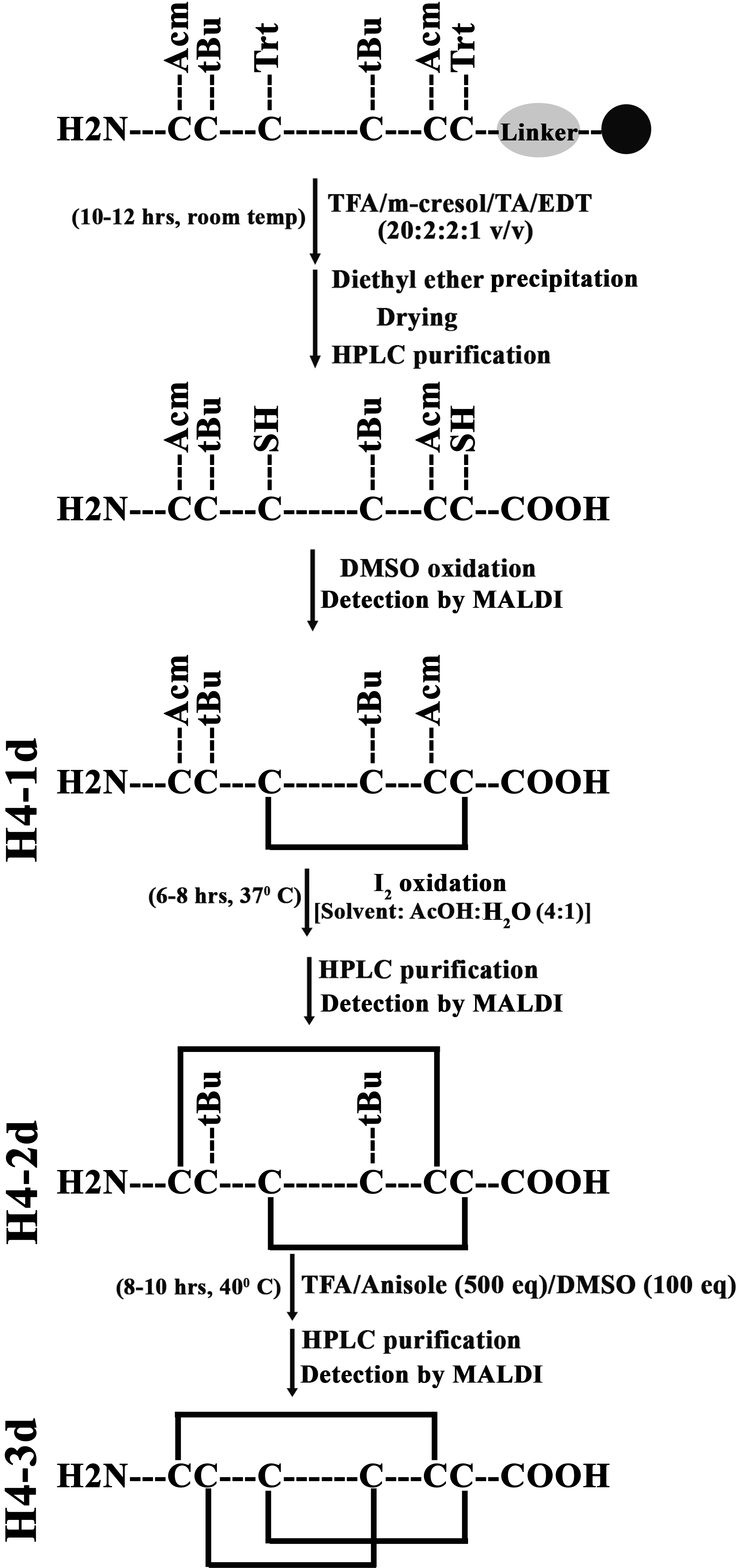

Supplement: S1 Fig — Formation of three disulfide linkages in HBD4 analogs H4-1d, H4-2d and H4-3d with regioselective strategy. Keys: trityl (Trt), acetamidomethyl (Acm), tertiary-butyl (tBu), ethanedithiol (EDT), thioanisole (TA), acetic acid (AcOH.) (TIF) [file pone.0119525.s001.tif]

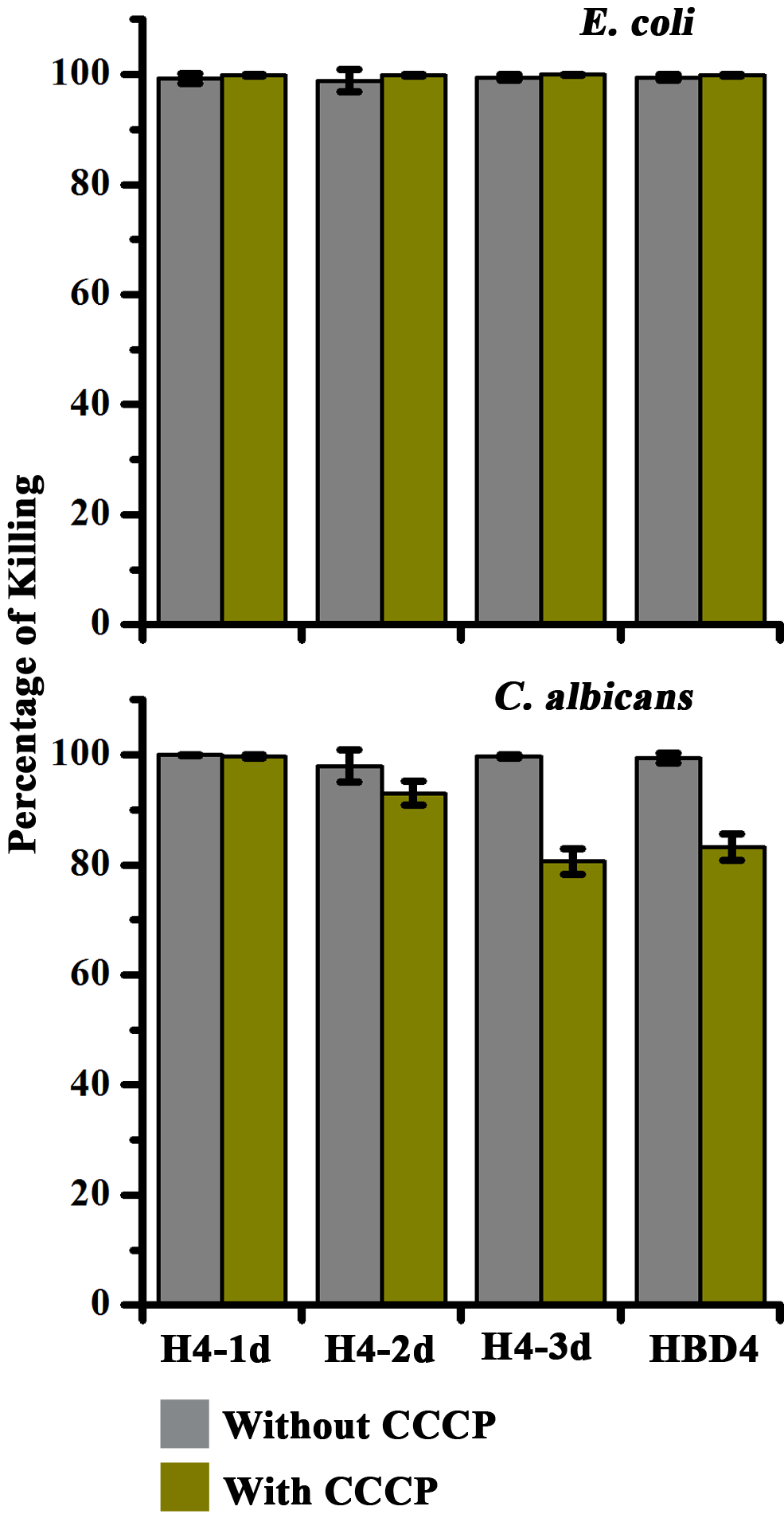

Supplement: S2 Fig — Antibacterial and antifungal activity of H4-1d, H4-2d, H4-3d and HBD4 in the presence and absence of CCCP. The values are expressed as average of three independent experiments and the error bars represent standard deviations (range = 0.10–2.96). (TIF) [file pone.0119525.s002.tif]

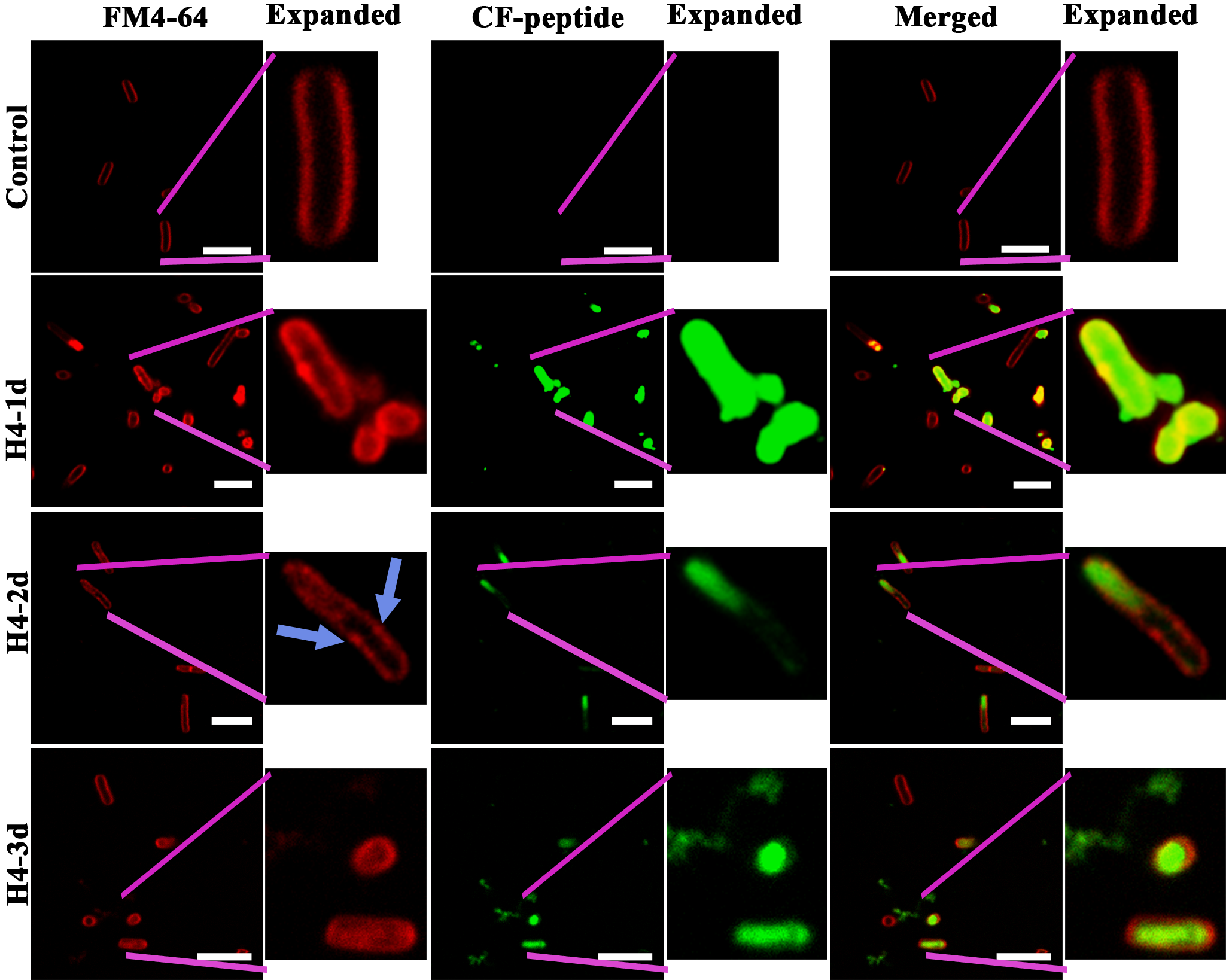

Supplement: S3 Fig — Localization of carboxyfluorescein (CF) labelled peptides H4-1d, H4-2d and H4-3d in P. aeruginosa stained with inner-memabrane dye FM4-64. Expanded images for bacteria are also shown adjacent to each panel indicated by pink lines. Blue arrows in FM4-64 expanded panel show membrane protrusion and lipid aggregation due to H4-2d. Scale bars represent 7.5 μm. (TIF) [file pone.0119525.s003.tif]
